# Motor Task-Dependent Dissociated Effects of Transcranial Random Noise Stimulation in a Finger-Tapping Task Versus a Go/No-Go Task on Corticospinal Excitability and Task Performance

**DOI:** 10.3389/fnins.2019.00161

**Published:** 2019-02-27

**Authors:** Andreas Jooss, Linus Haberbosch, Arvid Köhn, Maria Rönnefarth, Rouven Bathe-Peters, Leonard Kozarzewski, Robert Fleischmann, Michael Scholz, Sein Schmidt, Stephan A. Brandt

**Affiliations:** ^1^Department of Neurology, Charité – Universitätsmedizin Berlin, Berlin, Germany; ^2^Department of Neurology, Universitätsmedizin Greifswald, Greifswald, Germany; ^3^Neural Information Processing Group, Technische Universität Berlin, Berlin, Germany

**Keywords:** random noise stimulation, transcranial electrical stimulation, task dependency, finger-tapping task, go/no-go task, corticospinal excitability, neuroplasticity

## Abstract

**Background and Objective:** Transcranial random noise stimulation (tRNS) is an emerging non-invasive brain stimulation technique to modulate brain function, with previous studies highlighting its considerable benefits in therapeutic stimulation of the motor system. However, high variability of results and bidirectional task-dependent effects limit more widespread clinical application. Task dependency largely results from a lack of understanding of the interaction between externally applied tRNS and the endogenous state of neural activity during stimulation. Hence, the aim of this study was to investigate the task dependency of tRNS-induced neuromodulation in the motor system using a finger-tapping task (FT) versus a go/no-go task (GNG). We hypothesized that the tasks would modulate tRNS’ effects on corticospinal excitability (CSE) and task performance in opposite directions.

**Methods:** Thirty healthy subjects received 10 min of tRNS of the dominant primary motor cortex in a double-blind, sham-controlled study design. tRNS was applied during two well-established tasks tied to diverging brain states. Accordingly, participants were randomly assigned to two equally-sized groups: the first group performed a simple motor training task (FT task), known primarily to increase CSE, while the second group performed an inhibitory control task (go/no-go task) associated with inhibition of CSE. To establish task-dependent effects of tRNS, CSE was evaluated prior to- and after stimulation with navigated transcranial magnetic stimulation.

**Results:** In an ‘activating’ motor task, tRNS during FT significantly facilitated CSE. FT task performance improvements, shown by training-related reductions in intertap intervals and increased number of finger taps, were similar for both tRNS and sham stimulation. In an ‘inhibitory’ motor task, tRNS during GNG left CSE unchanged while inhibitory control was enhanced as shown by slowed reaction times and enhanced task accuracy during and after stimulation.

**Conclusion:** We provide evidence that tRNS-induced neuromodulatory effects are task-dependent and that resulting enhancements are specific to the underlying task-dependent brain state. While mechanisms underlying this effect require further investigation, these findings highlight the potential of tRNS in enhancing task-dependent brain states to modulate human behavior.

## Introduction

Transcranial electrical stimulation applied to the primary motor cortex is a non-invasive, portable, and low-cost method shown to enhance motor function in healthy subjects and maximize recovery after stroke (Talelli and Rothwell, 2006; Hummel et al., 2008). In addition to tDCS, tRNS is emerging as a promising neuromodulatory tool (Terney et al., 2008; Schmidt et al., 2013b; Prichard et al., 2014). In contrast to the constant direct current of tDCS, tRNS uses a biphasic alternating current with a random amplitude and frequency, drawn from a frequency range between 0.1–640 Hz (full spectrum) or 100–640 Hz (high-frequency). While tDCS modulates resting membrane potential, tRNS is understood to facilitate transmission of existing subthreshold neural activity to increase neuron excitability (Terney et al., 2008; Schmidt et al., 2013b).

Transcranial random noise stimulation is reported to provide considerable benefits over tDCS including polarity independence of stimulation effects (Terney et al., 2008), more pronounced effect sizes (Fertonani et al., 2011) and possibly improved reliability (Antal et al., 2010). Interestingly, tRNS has been suggested to be a vital component in a patterned, individualized stimulation algorithm aiming to maximize recovery after stroke (Schmidt et al., 2013b). Together, these findings suggest that tRNS might be more reliable, safer and better suited for therapeutic stimulation of the motor system.

However, a major and largely unresolved challenge across all transcranial electrical stimulation methods is the high variability of results, limiting more widespread clinical application. Important factors influencing interindividual variability in transcranial electrical stimulation studies are the baseline neuronal level of motor and cognitive function, psychological factors, circadian rhythm, genetics, anatomy, age, and variability in assessment methods (e.g., TMS) (Li et al., 2015). Additionally, since the state of neuron populations during stimulation is likely to play a pivotal role for the final behavioral effect, a significant part of variability is understood to be related to the brain’s task dependent activity state during stimulation (Silvanto et al., 2008; Li et al., 2015). The term brain state is used to describe characteristic changes in global brain activity dynamically adjusted to task demands (Gilbert and Sigman, 2007; Lee and Dan, 2012). Task dependency is a well-established phenomenon in non-invasive brain stimulation studies (Antal et al., 2007; Silvanto et al., 2008; Terney et al., 2008). It implies that the neuromodulatory effects of non-invasive brain stimulation might vary strongly dependent on the endogenous brain state both prior to as well as during stimulation.

In the motor system, CSE, acquired by TMS, is an electrophysiological parameter providing a direct, temporally and spatially precise readout to monitor task-dependent activation and inhibition via MEPs. CSE quantifies state changes of the stimulated motor cortex by probing post-synaptic corticospinal projections (Bestmann and Krakauer, 2015).

Studies aiming to modulate CSE and induce behavioral changes with tRNS highlight the controversial role of task-dependent brain states. tRNS was shown to have bidirectional task-dependent effects on CSE, which is associated with motor learning and recovery. tRNS applied *offline*, i.e., in idle subjects, was shown to increase CSE (Terney et al., 2008). Motor and cognitive tasks carried out *online*, i.e., during stimulation were shown to reduce CSE (Terney et al., 2008). Nevertheless, motor skill learning enhancements were found to be driven primarily by online effects during stimulation (Prichard et al., 2014). Saiote and colleagues investigated functional magnetic resonance imaging changes following a visuomotor task with online tRNS and found stimulation related blood-oxygen-level dependent changes only in regions related to the task, implying direct interaction of online tRNS with task related activity (Saiote et al., 2013). Results from these and other studies conducted in the visual- and cognitive domains (Fertonani et al., 2011; Pirulli et al., 2013; Snowball et al., 2013) suggest that the neuromodulatory effects of tRNS are dependent on *whether* a task and *what type* of task is performed online during stimulation, with enhancements specific to the engaged neural population or brain state.

The aim of this study was to investigate the task dependency of tRNS-induced neuromodulation in the motor system. The hypothesis of this study was that tRNS would modulate task effects in opposite directions, depending on the underlying brain state. Hence, for tRNS during a simple motor training task (FT task), known primarily to increase CSE, we hypothesize an increase in CSE and behavioral performance (Koeneke et al., 2006). For tRNS during an inhibitory control task (GNG task), associated with inhibition of CSE, we hypothesize a decrease in CSE and enhanced behavioral performance reflecting greater inhibition (Bestmann and Duque, 2016).

For this purpose, we closely monitored online as well as offline changes of behavioral and electrophysiological parameters that are established indicators of task-dependent brain states (Schmidt et al., 2013b). As the primary electrophysiological parameter, CSE was acquired via MEPs by nTMS. Compared to conventional, non-navigated TMS, nTMS uses an optical tracking system to control the physical variance related to the 3D parameters of the TMS coil in space. Since small divergences in TMS coil location and orientation can lead to significant variance in CSE estimates, nTMS is an often neglected, but essential prerequisite to reliably quantify changes of task-dependent brain states (Schmidt et al., 2009). Understanding the interaction between tRNS and task-dependent brain activity is imperative for increasing reliability, repeatability, and ultimately, therapeutic usefulness of this emerging neuromodulatory technique.

## Materials and Methods

### Participants

Thirty healthy, right-handed individuals (18 females, mean age 22.8 ± 2.8 years) received tRNS as well as sham stimulation to the dominant (left) primary motor cortex. All participants were right handed as assessed with the Edinburgh handedness inventory. General exclusion criteria for non-invasive brain stimulation were applied (Brunoni et al., 2011). Specifically, none of the subjects had a history of neurological disease, including movement disorders or epilepsy (Brunoni et al., 2011). All participants gave written informed consent. The study was approved by the local ethics committee and adheres to the principles of good clinical practice of the Charité – Universitätsmedizin Berlin (“Grundsätze der Charité zur Sicherung guter wissenschaftlicher Praxis”), as well as “The Code of Ethics of the World Medical Association” (Declaration of Helsinki).

### Experimental Paradigm

**FIGURE 1:**
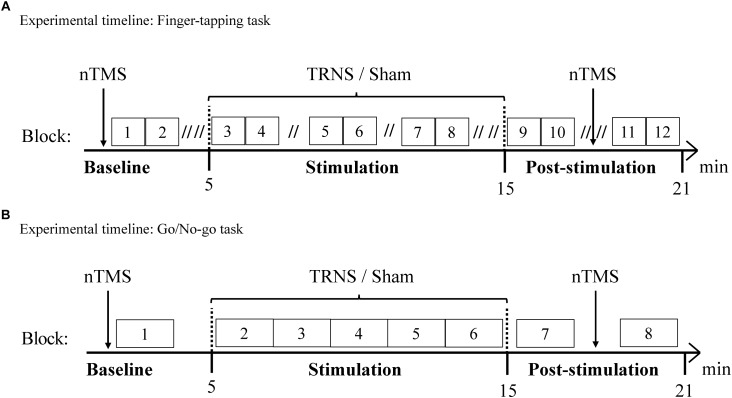
Experimental timelines. Behavioral measurements of the FT task **(A)** and the GNG task **(B)** were conducted along with nTMS to evaluate CSE. Behavioral and electrophysiological measurements were acquired in a baseline condition and a post-stimulation condition. Offline measurements were complemented by online behavioral assessments during 10 min stimulation with tRNS or sham stimulation. **(A)** Experimental timeline of the FT task. 15 participants performed the FT task. During one block of 60 s, one hand was tapping for 30 s before switching to the other hand for 30 s. Double slashes (“//”) denote a 60 s pause between blocks (“// //” = 120 s), to avoid excessive fatigue. **(B)** Experimental timeline of the GNG task. 15 participants performed the GNG task. One block consisted of 37 GNG trials and ended with a 15 s pause, resulting in 2 min per block.

A double-blind sham-controlled design was used in this study. The participants were randomly divided into two groups according to the task they were to perform during tRNS or sham stimulation: one group (15 participants) performed an ‘activating’ task (FT task) during stimulation, known primarily to increase CSE. The other 15 participants performed an ‘inhibitory’ task (GNG task), associated with inhibition of CSE. Behavioral and electrophysiological measurements were acquired offline in a baseline condition prior to stimulation, and a post-stimulation condition following 10 min of stimulation. Offline measurements were complemented by online behavioral assessments during stimulation as described below and in Figure 1. In this context, it is important to note that tasks served two functions during stimulation: they are indicators of task performance changes in response to stimulation and utilized to induce a well-established task-dependent brain state (Figure 1).

### Finger-Tapping Task (FT Task)

The experimental timeline for the FT task is depicted in Figure 1A. For the FT task, subjects were instructed to use the index finger of either hand to repeatedly exert a vertical force on a standard telegraph key as quickly and regularly as possible while receiving visual feedback on a screen. Visual feedback was provided with a live graphical display of ITIs on the x-axis and the corresponding number of taps on the y-axis. For the first block, the starting hand was randomly allocated and the tapping duration for one hand was 30 s before switching to the other hand for 30 s (Schulze et al., 2002). Two blocks for each hand (i.e., 4 × 30 s = 2 min) were followed by a 120 s pause (60 s pause during stimulation) to avoid excessive build-up of fatigue (Rönnefarth et al., 2018). As another precaution, the vertical force required to complete a tapping motion was adjusted to the lowest possible setting. Preventing excessive fatigue with regular pauses served to minimize its confounding influence on CSE (Terney et al., 2008). Prior to the experiment, participants were instructed and practiced the task for two blocks for each hand, resulting in a total of 1 min practice for each hand. The baseline condition consisted of two blocks for each hand, the stimulation condition (10 min) consisted of six blocks for each hand and the post-stimulation condition consisted of four blocks for each hand.

### Go/No-Go Task (GNG Task)

The experimental timeline for the GNG task is depicted in Figure 1B. One GNG trial with a total duration of 2.5–3 s followed the following time course: first, a fixation cross was presented on a screen, which lasted 1 s and was followed by a 250 ms warning cue (yellow square) (Joundi et al., 2012). Subsequently, a 250 ms target cue was presented with a varied latency of 250–750 ms based on an underlying, linearly increasing hazard rate, in line with (Schoffelen et al., 2005). Subjects exerted a maximal horizontal force on the lever only when a “go” cue (green circle) appeared (91%), while 9% of target cues were “no-go” cues (red circle) (Schoffelen et al., 2005). The hazard rate and the low probability of “no-go” trials were utilized to ensure optimal inhibition-related activity (Schoffelen et al., 2005; Wessel, 2018). The response period was limited to 750 ms.

During the GNG task, when no response was required, subjects maintained a horizontal isometric force of 4% of maximum voluntary contraction, with the index finger of the dominant hand on a lever, in line with (Kristeva et al., 2007). A low force output was used since it was shown to effectively enable corticospinal interaction and recruit most neurons in M1 (Evarts et al., 1983; Kristeva et al., 2007). The predetermined force was monitored throughout task execution and verbal feedback was given in case of deviations.

Prior to the experiment, participants were instructed and practiced 10 GNG trials. One block consisted of 37 GNG trials and a 15 s pause, resulting in 2 min per block. The baseline condition consisted of one block, the stimulation condition (10 min) consisted of five consecutive blocks (i.e., a total of 5 × 37 trials = 185 trials) and the post-stimulation conditions consisted of two blocks.

### Transcranial Random Noise Stimulation (tRNS)

Random noise stimulation was applied by a multi-channel low-voltage stimulation and EEG device certified for clinical use (NextWave, EBS Technologies GmbH, Kleinmachnow, Germany), which delivered weak random noise stimulation through conductive-rubber electrodes (NeuroConn GmbH, Ilmenau, Germany), placed in two saline-soaked sponges. One electrode (circular, 12.5 cm^2^) was situated over the dominant motor cortex at the C3 EEG electrode position (since all subjects were right-handed), the other electrode (rectangular electrode, 30 cm^2^) was placed over the contralateral frontopolar cortex (Moliadze et al., 2012). For tRNS, a peak-to-peak stimulation intensity of 1.51 mA (0.8 mA effective current intensity) was applied for 10 min with no DC offset. The random signal was drawn from a uniform probability density with a sample rate of 1280 Hz and digitally filtered to ensure a frequency distribution of 100–640 Hz, based on Terney et al. (2008). For sham stimulation, a 15 s ramp-up and 15 s ramp-down current was used in line with recommendations for tDCS (Nitsche et al., 2008; Schmidt et al., 2013a). Respective sessions of tRNS and sham stimulation were at least 7 days apart to avoid carry-over effects.

### Navigated Transcranial Magnetic Stimulation (nTMS)

Single pulse nTMS (eXimia VR TMS, Nexstim, Helsinki, Finland) with optical tracking and subject-specific magnetic resonance images was used in combination with a biphasic figure-of-eight coil (70-mm wing diameter) to evaluate CSE with optimal control of physical parameters (Schmidt et al., 2015). Compared to conventional, non-navigated TMS, nTMS was shown to reduce MEP amplitude variance by 27% (Schmidt et al., 2009). Electromyography activity in response to nTMS was recorded from the dominant first dorsal interosseus muscle with Neuroline 700 surface electrodes (Ambu VR, Ballerup, Denmark) arranged in belly-tendon montage. MEP amplitude was defined by peak-to-peak measurement. The stimulation target was the “center of gravity” of the dominant first dorsal interosseus (Wassermann et al., 1992). Resting motor threshold was defined as the stimulation intensity required to elicit a 500 μV MEP appearing with 50% probability using the maximum-likelihood threshold detection method and a 95% confidence interval, ensuring an individually calibrated intensity prior to data acquisition in each session (Awiszus, 2003). CSE was then assessed with 20 MEPs prior to and after electrical stimulation at the timepoints specified in Figure 1.

### Analysis and Statistics

Two subjects withdrew consent to participate in the study before completion. The remaining 28 subjects (13 in the FT group, 15 in the GNG group) were included in the analysis and statistics.

CSE data was manually reviewed and outliers, defined as values above or below 2.2x the interquartile range, were identified in each session and removed (Hoaglin and Iglewicz, 1987). CSE was estimated by using an in-house algorithm that accounted for physiological and physical confounders, such that MEPs associated with confounding prestimulus muscle contraction (preinnervation) above 20 μV and 100 ms prior to stimulation were excluded and further physical and physiological covariance was partitioned out of CSE estimation with step-wise regression (Schmidt et al., 2015). Mean CSE data was then baseline normalized by subtracting baseline values from post-stimulation values. Normality of data was graphically confirmed with histograms and by using the Shapiro–Wilk test. Levene’s test confirmed homogeneity of variances. Statistical analysis was conducted using a mixed model ANOVA to compare the main and interaction effects on CSE, with TASK (i.e., GNG, FT) as between-subjects factor and STIMULATION (i.e., tRNS, sham) as within-subjects factor.

Go/no-go task RTs, GNG task accuracy, FT ITI and FT taps were manually reviewed, which lead to exclusion of three subjects in the GNG group due to technical artifacts in the data. RTs and ITIs were outlier corrected, baseline normalized and z-transformed on a per subject basis over each session, in line with recommendations for within-subject designs and psychophysiological data (Bush et al., 1993). GNG accuracy data and FT taps were outlier corrected and baseline normalized for statistical analysis. Outlier correction involved trimming data by 5% of highest and lowest scores (Bush et al., 1993; Whelan, 2017). For GNG RTs specifically, trials without response and RTs below 100 ms after target cue presentation were rejected (Joundi et al., 2012). Baseline normalization required the mean of the baseline condition to be subtracted from the data. Z-transformation was used to increase power in comparison to raw means by accounting for intraindividual variability across subjects (Bush et al., 1993). A normal distribution could be confirmed both graphically as well as mathematically by the Shapiro–Wilk test. A linear mixed model for repeated measures was used to analyze the effect of tRNS on behavioral performance in the FT task and GNG task. It was used in favor of a repeated measures ANOVA due to its extended flexibility with regard to unbalanced data and precision in giving less biased estimates of fixed effects in repeated, correlated measurements (Cnaan et al., 1997; Krueger and Tian, 2004). As fixed effects, STIMULATION (i.e., tRNS/sham) and TIME (i.e., block) was entered into the model. SUBJECTS was entered as random effects. For a significant interaction of STIMULATION × TIME, *post hoc* tests for individual blocks were controlled for multiple comparisons using Bonferroni correction.

All digital signal processing was carried out with custom-made scripts within the MATLAB programming environment (MATLAB R2014a, The MathWorks, Inc., Natick, MA, United States). All statistical analysis was performed using SPSS Statistics with statistical significance level set at α = 0.05 (IBM SPSS Statistics for Windows, Version 21.0. Armonk, NY, United States: IBM, Corp.). Results are presented as mean values and standard errors of the mean unless stated otherwise.

## Results

### Corticospinal Excitability (CSE)

**FIGURE 2:**
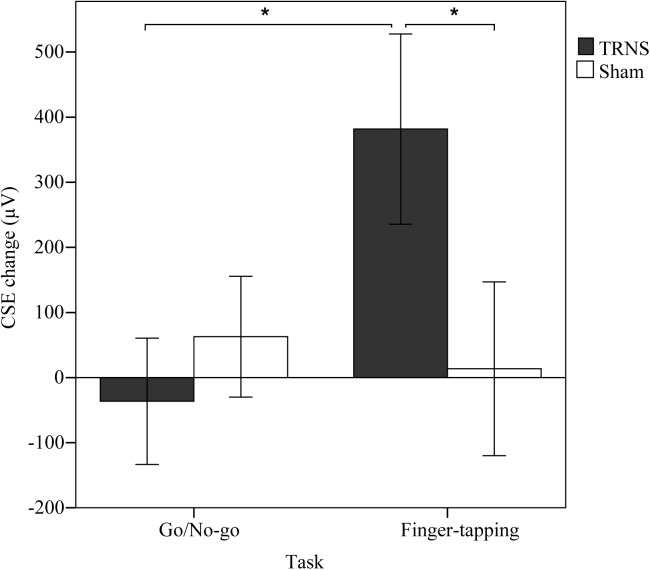
Effects of tRNS on corticospinal excitability. Mean CSE change (μV) was calculated by subtracting baseline CSE measurements from post-stimulation measurements. CSE change is depicted for respective task type (GNG or FT) performed during 10 min of stimulation with either tRNS or sham stimulation. Error bars depict the standard error of the mean. In the FT group, MEP responses were significantly facilitated (^∗^) after tRNS compared to sham stimulation and tRNS in the GNG group.

Effects of tRNS on CSE are depicted in Figure 2. Mean uncorrected baseline CSE for the FT group was similar for the tRNS (691 ± 89 μV) and the sham condition (FT, sham: 686 ± 125 μV) [*t*(12) = 9.032, *p* = 0.975]. Baseline CSE for the GNG group was also similar for the tRNS (530 ± 71 μV) and the sham condition (500 ± 105 μV) [*t*(14) = 0.250, *p* = 0.806]. In the mixed model ANOVA, there was no significant main effect of TASK [*F*(1,26) = 1.961, *p* = 0.173, $\eta_{p}^{2}$ = 0.07] and STIMULATION [*F*(1,26) = 1.814, *p* = 0.19, $\eta_{p}^{2}$ = 0.05] on CSE. However, there was a significance for the interaction STIMULATION × TASK [*F*(1,26) = 5.474, *p* = 0.027, $\eta_{p}^{2}$ = 0.17], indicating that excitability changes were dependent on the specific stimulation applied during task execution. Pairwise comparisons revealed that in the FT group, baseline corrected MEP responses were significantly facilitated following tRNS (381 ± 146 μV) compared to sham stimulation (14 ± 133 μV) (*p* = 0.018, $\eta_{p}^{2}$ = 0.2). In the GNG group, tRNS (-36 ± 97 μV) did not influence MEP responses compared to sham stimulation (-63 ± 93 μV) (*p* = 0.473, $\eta_{p}^{2}$ = 0.02). This shows that tRNS specifically increased CSE after the FT task but not after the GNG task (*p* = 0.022, $\eta_{p}^{2}$ = 0.19) (Figure 2).

### FT: Intertap Interval (ITI)

**FIGURE 3:**
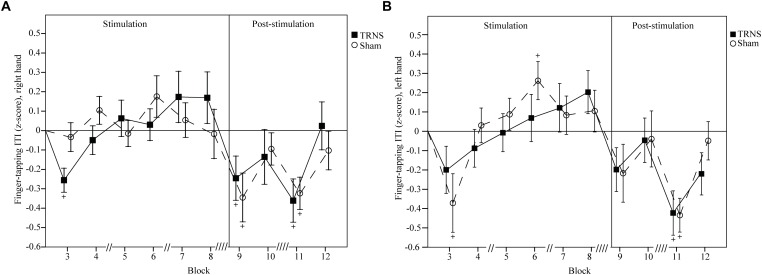
Effects of tRNS on FT ITI. Mean FT ITIs are baseline corrected and z-transformed. Blocks 3–8 (30 s per block) depict ITIs during electrical stimulation, while blocks 9–12 present data post-stimulation. Double slashes (“//”) denote a 60 s pause between blocks (“// //” = 120 s), to avoid excessive fatigue. Mean ITI is displayed with standard error of the mean. Significant changes from baseline are marked with “+.” ITIs of the right hand **(A)** and the left hand **(B)** were not significantly different between the tRNS condition compared to the sham condition. For both hands, singular significant reductions in ITIs in block 3 of one condition likely represent a rebound effect after a prior pause. Reductions in ITIs post-stimulation for both the tRNS and sham conditions imply motor learning.

Effects of tRNS on FT ITIs are depicted in Figure 3A (right hand) and Figure 3B (left hand). Uncorrected baseline ITIs were shorter for the right hand (tRNS, 148 ± 6 ms; sham, 149 ± 5 ms) compared to the left hand (tRNS, 170 ± 6 ms; sham, 170 ± 6 ms).

For the right hand, a linear mixed model did not show a significant main effect of STIMULATION on FT ITIs [*F*(2) = 2.35, *p* = 0.6]. However, a significant interaction of STIMULATION × TIME could be observed [*F*(20) = 3.03, *p* < 0.001]. *Post hoc* tests revealed significant reductions in ITIs after both tRNS (block 9, -0.246 ± 0.104, *p* = 0.02; block 11, -0.361 ± 0.101, *p* < 0.001) and sham stimulation (block 9, -0.345 ± 0.105, *p* = 0.001; block 11, -0.323 ± 0.101, *p* = 0.001). ITIs at the beginning of stimulation in block 3 were significantly faster only in the tRNS condition (-0.256 ± 0.101, *p* = 0.012). Bonferroni corrected pairwise comparisons between individual blocks and stimulation did not reach significant results (Figure 3A).

For the left hand, a linear mixed model did not show a significant main effect of STIMULATION on FT ITIs [*F*(2) = 2.86, *p* = 0.58]. However, a significant interaction of STIMULATION × TIME could be observed [*F*(20) = 3.29, *p* < 0.001]. *Post hoc* tests revealed significant reductions in ITIs after both tRNS (block 11, -0.423 ± 0.117, *p* < 0.001) and sham stimulation (block 3, -0.371 ± 0.112, *p* = 0.001; block 11, -0.434 ± 0.112, *p* < 0.001). There was a significant increase in ITIs the sham condition in block 6 (0.262 ± 0.112, *p* = 0.02) during stimulation. Bonferroni corrected pairwise comparisons between individual blocks and stimulation did not reach significant results (Figure 3B).

### FT: Finger Taps

**FIGURE 4:**
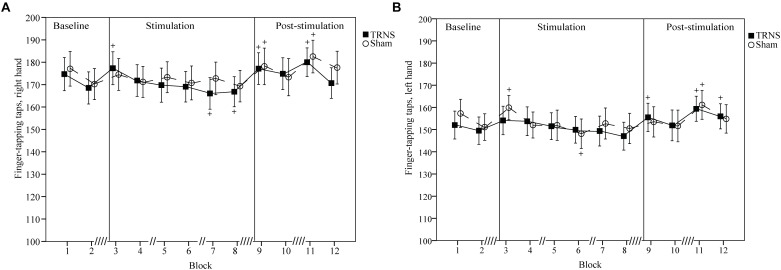
Effects of tRNS on FT taps. Mean FT number of taps are shown which illustrate an overall higher tapping performance of the right hand **(A)** compared to the left hand **(B)** and complement changes in FT ITIs observed in Figure 3. Blocks 3–8 (30 s per block) depict finger taps during electrical stimulation, while blocks 9–12 present data post-stimulation. Double slashes (“//”) denote a 60 s pause between blocks (“// //” = 120 s), to avoid excessive fatigue. Mean finger taps are displayed with standard error of the mean. Significant changes from baseline are marked with “+.” **(A,B)** Number of finger taps for both hands were not significantly different between the tRNS condition compared to the sham condition. For both hands, singular significant increases in the number of finger taps in block 3 of one condition likely represent a rebound effect after a prior pause. Significant reductions during stimulation represent fatigue. Increased number of finger taps post-stimulation for both tRNS and sham conditions imply motor learning.

Effects of tRNS on FT taps are depicted in Figure 4A (right hand) and Figure 4B (left hand). Mean uncorrected baseline finger taps were higher for the right hand (tRNS, 171.62 ± 7.18; sham 173.65 ± 7.32) compared to the left hand (tRNS 150.81 ± 6.17; sham 154.23 ± 6.11).

For the right hand, a linear mixed model with baseline corrected data did not show a significant main effect of STIMULATION on FT taps [*F*(2) = 1.98, *p* = 0.14]. However, a significant interaction of STIMULATION × TIME could be observed [*F*(20) = 3.39, *p* < 0.001]. *Post hoc* tests revealed significant increases in the number of finger taps versus baseline for tRNS (block 3, 5.69 ± 2.34, *p* = 0.016; block 9, 5.54 ± 2.34, *p* = 0.019; block 11, 8.38 ± 2.34, *p* < 0.001) and sham stimulation (block 9, 7 ± 2.44, *p* = 0.004; block 11, 8.88 ± 2.33, *p* < 0.001). Additionally, toward the end of tRNS, the number of finger taps was significantly reduced versus baseline (block 7, -5.54 ± 2.34, *p* = 0.019; block 8, -4.77 ± 2.34, *p* = 0.043). Bonferroni corrected pairwise comparisons between individual blocks and stimulation did not reach significant results (Figure 4A).

For the left hand, a linear mixed model with baseline corrected data showed a significant main effect of STIMULATION on FT finger taps [*F*(2) = 3.45, *p* = 0.03] with a significant increase in FT finger tap estimates of fixed effects for tRNS (2.06 ± 0.79) [*t*(255) = -2.62, *p* = 0.09] but not for sham (0.16 ± 0.8) [*t*(255) = 0.2, *p* = 0.84]. However, *post hoc* tests between tRNS and sham did not reveal a significant difference between stimulation conditions [*t*(255) = -1.7, *p* = 0.09]. A significant interaction of STIMULATION × TIME could be observed [*F*(20) = 2.63, *p* < 0.001]. *Post hoc* tests revealed significant increases in the number of finger taps versus baseline after both tRNS (block 9, 4.73 ± 2.37, *p* = 0.047; block 11, 8.58 ± 2.37, *p* < 0.001; block 12, 5.69 ± 2.34, *p* = 0.029) and sham stimulation (block 3, 5.69 ± 2.37, *p* = 0.017; block 11, 6.92 ± 2.37, *p* = 0.004). Additionally, toward the end of sham stimulation, the number of finger taps was significantly reduced versus baseline (block 6, -6.08 ± 2.37, *p* = 0.011). Bonferroni corrected pairwise comparisons between individual blocks and stimulation did not reach significant results (Figure 4B).

### GNG: Reaction Time (RT)

**FIGURE 5:**
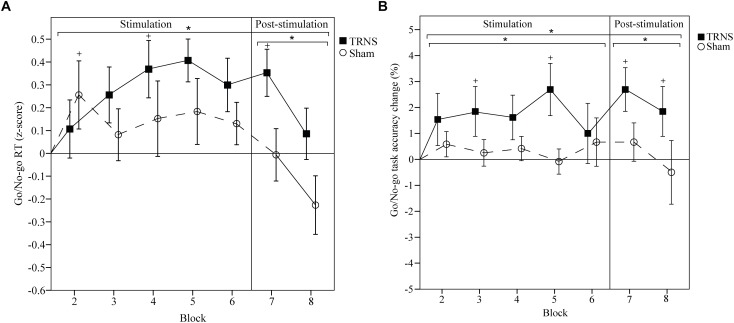
Effects of tRNS on GNG RT and task accuracy. **(A,B)** Mean GNG RT and task accuracy are baseline corrected. RTs are z-transformed. Blocks 2–6 (2 min per block) depict RTs and task accuracy change during electrical stimulation, while blocks 7 and 8 present data post-stimulation. Means are displayed with standard error of the mean. Significant changes from baseline are marked with “+.” Significant changes compared to sham are marked with “^∗^.” **(A)** RTs were significantly longer in the tRNS condition compared to sham. **(B)** Task accuracy was significantly improved during and after tRNS compared to sham. Together, these results suggest that tRNS specifically strengthened motor inhibition and inhibitory control in the GNG task.

Effects of tRNS on GNG RT are depicted in Figure 5A. Mean uncorrected baseline RT for the tRNS condition was 303 ± 5 ms, and 313 ± 7 ms for the sham condition. A linear mixed model showed a significant main effect of STIMULATION on GNG RTs [*F*(2) = 11.69, *p* < 0.001] with a significant increase in estimates of fixed effects for tRNS (0.21 ± 0.045) [*t*(160) = 4.65, *p* < 0.001] but not for sham (0.06 ± 0.045) [*t*(160) = 1.33, *p* = 0.19]. Importantly, *post hoc* tests between tRNS and sham revealed a significant difference between stimulation conditions [*t*(160) = -2.35, *p* = 0.019]. Breaking down the main effect of STIMULATION into a stimulation period (blocks 2–6) and a post-stimulation period (blocks 7–8), the linear mixed model for RTs post-stimulation was significant [*F*(2) = 5.48, *p* = 0.007], with a significant difference in estimates of fixed effects: GNG RTs were attenuated after tRNS (0.24 ± 0.085, *p* = 0.06) compared to sham (-0.14 ± 0.085, *p* = 0.107) [*t*(44) = -3.19, *p* = 0.002]. There was no significant difference between tRNS and sham during the stimulation period [*t*(114) = -0.79, *p* = 0.43). A significant interaction of STIMULATION × TIME could also be observed [*F*(14) = 2.57, *p* = 0.002]. *Post hoc* tests showed attenuated RTs for tRNS in block 5 (0.284 ± 0.122, *p* = 0.021) and block 7 (0.32 ± 0.122, *p* = 0.01) and at the start of sham stimulation (block 2; 0.256, ± 0.117, *p* = 0.031). Bonferroni corrected pairwise comparisons between individual blocks and stimulation did not reach significant results. Together, these results show that tRNS specifically attenuated RTs in the GNG task in the post-stimulation period (Figure 5A).

### GNG: Task Accuracy

Effects of tRNS on GNG task accuracy are depicted in Figure 5B. Mean uncorrected baseline GNG task accuracy for the tRNS condition was 96.88 ± 0.91 and 98.34 ± 0.58% for the sham condition. A linear mixed model showed a significant main effect of STIMULATION on baseline corrected GNG accuracy [*F*(2) = 18.01, *p* < 0.001] with a significant increase in estimates of fixed effects for tRNS (1.89 ± 0.32) [*t*(173) = 5.94, *p* < 0.001] but not for sham (0.29 ± 0.33) [*t*(173) = 0.86, *p* = 0.39]. Importantly, *post hoc* tests between tRNS and sham revealed a significant difference between stimulation conditions [*t*(173) = -3.49, *p* < 0.001]. Breaking down the main effect of STIMULATION into a stimulation period (blocks 2–6) and a post-stimulation period (blocks 7–8), the linear mixed model for GNG accuracy during stimulation was significant [*F*(2) = 12, *p* < 0.001], with a significant difference in estimates of fixed effects: GNG accuracy was increased during tRNS (1.74 ± 0.36, *p* < 0.001) compared to sham (0.37 ± 0.38, *p* = 0.973) [*t*(123) = -2.62, *p* = 0.009]. GNG accuracy was also significantly increased in the post-stimulation period [*F*(2) = 5.97, *p* = 0.005] with significant differences in estimates of fixed effects after tRNS (2.27 ± 0.66, *p* = 0.001) compared to sham (0.083 ± 0.68, *p* = 0.904) [*t*(48) = -2.30, *p* = 0.023). A significant interaction of STIMULATION × TIME could also be observed [*F*(14) = 2.78, *p* = 0.001]. *Post hoc* tests showed increased task accuracy during tRNS in block 3 (1.85 ± 0.86, *p* = 0.033), block 5 (2.69 ± 0.86, *p* = 0.002) and after tRNS in block 7 (2.69 ± 0.86, *p* = 0.002) and block 8 (1.85 ± 0.86, *p* = 0.033). The sham condition did not reach significant results. Bonferroni corrected pairwise comparisons between individual blocks and stimulation did not reach significant results. Together, these results show that tRNS specifically increased task accuracy in the GNG task during stimulation and in the post-stimulation period (Figure 5B).

## Discussion

The purpose of this study was to investigate the task dependency of tRNS-induced neuromodulation in the motor system. The main results of this study show task-dependent dissociated effects on CSE and behavioral performance following tRNS during a FT task versus a GNG task. After motor training (FT task), characterized by repetitive motor activation, tRNS led to significant facilitation of CSE compared to sham stimulation, while behavioral performance was not significantly different to sham stimulation. Conversely, in the inhibitory control task (GNG task), tRNS-enhanced inhibition led to an attenuation of RTs without effects on CSE. Together, these findings support the notion that tRNS enhances the predominant task-dependent brain state. Our results highlight the interaction between tRNS and task-dependent brain activity and provide further evidence for tRNS’ proposed mechanisms of action.

### Motor Activation

In the simple motor training task (FT), online tRNS significantly facilitated CSE as compared to sham stimulation. To our knowledge, we are the first to show CSE enhancements after task execution during tRNS. CSE enhancements after tRNS have been previously shown only in idling subjects. In idling subjects, reliable CSE increases lasting 60 min are possible (Terney et al., 2008). Additionally, with regards to tRNS parameters, high frequency tRNS (100–640 Hz) (Terney et al., 2008) at high current intensities (1 mA) (Moliadze et al., 2012) with a duration of at least 5 min (Chaieb et al., 2011) was also shown to reliably increase CSE. In contrast, online tRNS was previously reported to impede CSE enhancements: CSE was found to be slightly attenuated for a cognitive task and strongly attenuated for a motor task (Terney et al., 2008). Attenuation after the motor task was suggested to be associated with task-induced fatigue (Terney et al., 2008).

Results from this study suggest that CSE facilitation after the FT task with online tRNS reflects an enhancement of task-dependent activation, i.e., additional motor activation in primed neural populations. Simple tapping tasks are well-established as prototype tasks to study motor training-induced neuroplasticity in the primary motor cortex (for a review see, Ljubisavljevic, 2006; Bezzola et al., 2012). Maximal sequential movements of the FDI ensure a maximum task-related activation of its cortical representation in M1, minimizing a confounding influence from other brain areas (Bezzola et al., 2012). Motor activation is independent of the physical tapping speed of subjects, since the amount of neural effort determines maximal neurophysiological activation (Lutz et al., 2005). Motor training leads to larger muscle representations, specific to the muscles involved in the task, and increased CSE (Pascual-Leone et al., 1994; Muellbacher et al., 2001; Koeneke et al., 2006).

The observation of further enhancement of task-dependent activation with tRNS fits well in line with the current understanding of tRNS’ proposed mechanism of action: increase of CSE via transmission of subthreshold neural signals – a phenomenon known as stochastic resonance (Terney et al., 2008). Stochastic resonance, i.e., the mechanism by which an optimal noise condition improves signal detection in non-linear systems, has been known in the physics community since at least the early 1980s and has been universally observed in various neural systems including the human brain (Moss et al., 2004; Schmidt et al., 2013b).

Unchanged CSE levels after sham stimulation suggest that the tapping training duration was not sufficiently long to increase functional recruitment in the absence of tRNS. Motor training studies typically last 30–60 min (Classen et al., 1998; Muellbacher et al., 2001; Koeneke et al., 2006). These studies highlight the crucial addition of online tRNS in our study to dramatically reduce the required time for motor training-induced neuroplasticity in the primary motor cortex.

### Motor Fatigue and Motor Learning

The FT task is a simple motor training task involving motor fatigue and motor learning indexed by a change in ITIs. It has been utilized as a clinical tool to characterize motor deficits in Parkinson’s disease, cerebellar dysfunction, stroke and as a result of aging (Shimoyama et al., 1990; Arias et al., 2012).

In the present study, a linear increase in mean ITIs and finger taps *during* electrical- and sham stimulation represents task-induced motor fatigue (Rönnefarth et al., 2018). Fatigue inevitably occurs within seconds of task initiation (Shimoyama et al., 1990; Aoki et al., 2003; Rönnefarth et al., 2018). It involves not only peripheral, but also central mechanisms (central motor fatigue) as evidenced by reduced CSE after a fatiguing task (Kluger et al., 2012). Therefore, fatigue is a potential confounder in brain stimulation studies aiming to enhance CSE levels and likely explains CSE disruptions previously observed after online tRNS in the motor system (Antal et al., 2007; Terney et al., 2008). Several measures were taken in our study to tune the FT task to reduce the influence of fatigue (see section “Finger-Tapping Task (FT Task)). These measures were effective in preventing fatigue outlasting the stimulation condition, since post-stimulation ITIs and finger taps were equal to or lower than baseline levels and CSE inhibition, typically seen after excessive fatigue, was absent. Reduced ITIs and increased number of finger taps compared to baseline in block 3 (right hand), at the beginning of tRNS were not significant compared to sham stimulation and likely represent a rebound effect after a prior pause of 120 s. This might also explain the analogous phenomenon in block 3 of the left hand, at the beginning of sham stimulation.

The significant ITI enhancements and increased number of finger taps *after* tRNS and sham stimulation (between blocks 9–12) show that the utilized FT task was efficient in inducing motor learning. These unspecific effects on motor learning gain special significance when interpreted with corresponding CSE results: although the FT task improvements in the right hand were also observed in the sham condition, facilitation of CSE occurred only after tRNS. This implies that electrical stimulation might be associated with an enhanced potential for learning (Koeneke et al., 2006). Motor learning is known to occur as a result of motor training (for a review see, Ljubisavljevic, 2006), and to be closely associated with CSE facilitation and ITI improvements in simple tapping tasks (Koeneke et al., 2006). Further studies also emphasize the robust relation between motor learning and excitability enhancements, e.g., CSE levels return to baseline once subjects overlearn a task (Muellbacher et al., 2001) and improvement retention is disrupted when CSE is specifically suppressed over M1 (Muellbacher et al., 2002).

The robust behavioral improvements in the FT task after stimulation could not be differentiated (i.e., tRNS, sham), possibly due to a ceiling effect. In the young, healthy participants of this study, underlying motor learning processes are likely to be already optimized. Additionally, maximum task-related activation of M1 is thought to leave no room for further performance gains, especially in early stages of motor learning (Bezzola et al., 2012). Other measures of FT task performance, e.g., force and tapping duration might expose tRNS-specific behavioral gains with higher sensitivity (Muellbacher et al., 2001; Rönnefarth et al., 2018). Providing evidence for neuromodulation of motor learning would be particularly relevant in the context of novel interventions following brain injury (Pascual-Leone et al., 2005).

### Motor Inhibition

Unlike the simple motor training task, random noise stimulation in the inhibitory control task (GNG task) left CSE unchanged in both the tRNS and sham conditions, suggestive of an underlying inhibitory task-dependent brain state counteracting the facilitatory tRNS effects reported in idle subjects (Terney et al., 2008). We hypothesized a decrease in CSE after GNG and tRNS, reflecting enhanced motor inhibition. Methodological limitations and task complexity might have contributed to the absence of a clearer MEP decrease:

Firstly, CSE measurements after tRNS were not obtained on a trial-by-trial basis during GNG task execution and do not trace the time course of transient inhibitory state fluctuations per trial. The GNG task is a hallmark for motor inhibition encompassing periods of response preparation and response inhibition reflected by changes in CSE, for a review see Greenhouse et al. (2015) and Bestmann and Duque (2016). As subjects engage in the task and prepare to respond, motor inhibition, characterized by reduced MEPs, prevents a premature response (Greenhouse et al., 2015). The warning cue further enhances inhibitory processes (Boulinguez et al., 2009; Criaud et al., 2012) and the specificity of suppression to the muscles involved in the task (Greenhouse et al., 2012). If a “no-go” target cue appears, response inhibition acts as an active breaking process leading to global suppression of motor cortical activity with concurrent MEP suppression (Stinear et al., 2009; Greenhouse et al., 2012; MacDonald et al., 2014; Bestmann and Duque, 2016). Since CSE was investigated with single pulse nTMS *after* task execution, any potential transient enhancement of motor inhibition *during* the GNG task would not be detected in our paradigm.

Secondly, inhibition is interrupted by “go” cues requiring motor activation with concurrent brief facilitation of CSE (Stinear et al., 2009; MacDonald et al., 2014). These short but frequent motor responses might have contributed to the absence of a clear MEP suppression. Yet, rare “no-go” trials (<20%) are required to ensure sufficient inhibition-related activity and a 9% “no-go” probability has been shown to induce such activity (Schoffelen et al., 2005; Wessel, 2018). As becomes apparent, the inhibitory state associated with the GNG task is comparably more complex than the FT task. It includes the subcomponents response preparation, response inhibition, response activation and poses the methodological challenge of tracking these dynamically overlapping state changes with sufficient temporal resolution.

### Inhibitory Control

Considering limitations arising from using single pulse nTMS to measure CSE after task completion, RT and task accuracy data acquired online, during the GNG task, serve as an easily assessable, more adequate parameter. RT and task accuracy are behaviorally relevant and trace dynamic state changes with a higher temporal resolution. RTs were significantly slowed in the tRNS condition, especially after electrical stimulation, while task accuracy was enhanced. Slowing of RTs in “go” trials is commonly used as a surrogate parameter for motor inhibition and is positively correlated to task accuracy (Bezdjian et al., 2009; Leotti and Wager, 2010). Response slowing is associated with suppression of MEPs, very similar to mechanisms involved in response inhibition (Jahfari et al., 2010).

The speed-accuracy trade-off is modulated by intraindividual inhibitory control: patients with impulse control disorders such as attention deficit and hyperactivity disorder (ADHD) and in patients who stutter, the speed-accuracy trade-off is shifted toward deficient inhibitory control with faster RTs and lower task accuracy (Bezdjian et al., 2009; Eggers et al., 2013). In turn, longer RTs and better task accuracy as signs of enhanced inhibitory control are achieved in patients with ADHD by pharmacological agents such as Modafinil (Turner et al., 2004). This phenomenon can likewise be observed in healthy subjects depending on gender (enhanced in female) and motivation (Bezdjian et al., 2009; Leotti and Wager, 2010). Consequently, we propose slowed RTs and enhanced task accuracy during and after tRNS to result from strengthened motor inhibition and inhibitory control outlasting stimulation. Our data suggests that tRNS impedes movement initiation by stabilizing the existing task-dependent brain state and delaying response initiation (Schmidt et al., 2013b). Future tRNS studies could try to modulate and optimize the speed-accuracy-tradeoff via task difficulty and in patients with deficient inhibitory control.

## Conclusion

We provide evidence that tRNS-induced neuromodulation in the motor system is dependent on the task during stimulation such that CSE is enhanced in a FT task and inhibitory control is improved in a GNG task. Results confirm our hypothesis that transcranially applied random noise stimulation enhances the endogenous task-dependent brain state of healthy subjects. To our knowledge, we are the first to show CSE facilitation after online tRNS during a FT task. We argue in favor of online tRNS to avoid contradictory results and expose task specific regulatory processes to be modulated by transcranial stimulation techniques. Further confirmation of tRNS’ mechanism of action is required to limit variability as a result of task dependency and to potentiate its neuroplastic effects in health and disease.

## Data Availability

The datasets generated for this study are available on request to the corresponding author.

## Author Contributions

SS, MS, and SB conceived the principle idea of the work. AJ, SS, MS, SB, LH, AK, MR, and RF designed the experiments. MS developed the software for experimental procedures and electrical stimulation. AJ, LH, and AK performed the measurements. AJ, SS, LH, LK, and RB-P conducted computational and statistical analyses of the data. All authors participated in the interpretation of the data. The manuscript was drafted by AJ and critically revised and approved by all authors.

## Conflict of Interest Statement

The authors declare that the research was conducted in the absence of any commercial or financial relationships that could be construed as a potential conflict of interest.

## Abbreviations

CSE: corticospinal excitability
FT: finger-tapping
GNG: go/no-go
ITI: intertap interval
MEP: motor evoked potential
nTMS: navigated transcranial magnetic stimulation
RT: reaction time
tDCS: transcranial direct current stimulation
TMS: transcranial magnetic stimulation
tRNS: transcranial random noise stimulation
